# The Oncogenic Role of Serum Marker GDF15 in Promoting Colorectal Tumorigenesis via EMT and Stemness

**DOI:** 10.1155/sci/4695395

**Published:** 2026-02-03

**Authors:** Hui Xu, Quancheng Zhang, Qing Li, Feng Gu, Duping Wang, Yiqing Tian

**Affiliations:** ^1^ Department of Ophthalmology, The First Affiliated Hospital of Anhui Medical University, Hefei, 230001, Anhui, China, ahmu.edu.cn; ^2^ Department of Blood Transfusion, The First Affiliated Hospital of Anhui Medical University, Hefei, 230001, Anhui, China, ahmu.edu.cn; ^3^ Department of Clinical Laboratory, Xuzhou Central Hospital, Xuzhou, 221009, Jiangsu, China, xzch.cn; ^4^ Department of Jiangsu Key Laboratory of Immunity and Metabolism, Xuzhou Medical University, Xuzhou, 221004, Jiangsu, China, xzmc.edu.cn

**Keywords:** colorectal cancer, EMT, GDF15, metastasis, proliferation, stemness

## Abstract

**Background:**

Growth Differentiation Factor 15 (GDF15), a stress‐responsive cytokine, is involved in the progression of various cancers. However, its precise functional role and underlying mechanism in colorectal cancer (CRC) remain unclear.

**Methods:**

GDF15 expression in CRC was analyzed using public databases and validated in patient tissues by Western blot. Functional assays, including colony formation, CCK‐8, wound‐healing, and Transwell, were performed on LOVO and HCT116 cells following GDF15 overexpression or knockdown to assess proliferation, migration, and invasion. Epithelial‐mesenchymal transition (EMT) and stemness markers were examined by Western blot. Cancer stem cell properties were evaluated using a tumorsphere formation assay.

**Results:**

GDF15 was significantly upregulated in CRC tissues at both mRNA and protein levels. In vitro, GDF15 overexpression in LOVO cells promoted proliferation, migration, and invasion and induced EMT, as evidenced by downregulated E‐cadherin and upregulated vimentin and N‐cadherin. Conversely, GDF15 knockdown in HCT116 cells produced opposite effects. Furthermore, GDF15 enhanced CRC cell stemness, increasing tumorsphere formation and upregulating stemness markers (CD133, SALL4, OCT4, NANOG). Clinically, high serum GDF15 levels were significantly associated with advanced age, late TNM stage, and elevated CEA, indicating its correlation with aggressive disease features.

**Conclusion:**

Our findings demonstrate that GDF15 acts as a tumor promoter in CRC by driving EMT, facilitating proliferation and metastasis, and enhancing cancer stemness. This study identifies GDF15 as a potential biomarker and therapeutic target for CRC.

## 1. Introduction

Colorectal cancer (CRC) is a leading cause of global cancer morbidity and mortality, ranking third in incidence and fourth in mortality worldwide [1, 2]. Despite advances in diagnostic and therapeutic strategies, the 5‐year survival rate for patients with advanced CRC remains poor, primarily due to metastasis [3]. Tumor metastasis remains the main cause of death for patients. Therefore, elucidating the molecular mechanisms underlying CRC progression and identifying novel diagnostic markers and therapeutic targets are crucial for improving patient outcomes.

Growth Differentiation Factor 15 (GDF15), also known as NAG‐1 (Non‐steroidal Anti‐inflammatory Drug‐activated Gene‐1), is a divergent member of the Transforming Growth Factor‐β (TGF‐β) superfamily [4]. It is synthesized as a 308‐amino‐acid precursor protein, which is proteolytically cleaved to release the mature bioactive peptide [5]. It serves as a crucial stress response cytokine, playing a key role in regulating diverse physiological and pathological processes, such as immune regulation, metabolic balance, and tumor development. Under physiological conditions, GDF15 is expressed at low levels in most tissues but is markedly induced in response to cellular stress, tissue injury, and inflammation [6–8]. GDF15 exerts its effects by binding to its specific receptor, GFRAL (glial cell line‐derived neurotrophic factor family receptor alpha‐like protein). GFRAL is mainly expressed in the last area of the medulla oblongata and the solitary nucleus [9, 10]. When the level of GDF15 exceeds the threshold, it triggers a series of physiological responses, including appetite suppression, nausea, and vomiting, as well as energy metabolism reprograming [11]. Beyond its metabolic functions, GDF15 exhibits immunomodulatory properties, such as inhibiting dendritic cell maturation to maintain fetal‐maternal tolerance during pregnancy [8]. The transcription of GDF15 is regulated by several transcription factors. Among them, p53 is one of the most important regulatory factors. When DNA is damaged, p53 can directly bind to the promoter region of GDF15 and induce its expression [12, 13]. In addition, transcription factors such as EGR1 also participate in the transcriptional regulation of GDF15 [14]. Consequently, GDF15 has been implicated in diverse pathophysiological processes, including obesity, insulin resistance, and fatty liver disease [15–18].

Emerging evidence suggests that GDF15 plays a context‐dependent role in cancer. It is frequently overexpressed in various malignancies and associated with poor prognosis. In CRC, elevated serum and tissue levels of GDF15 have been reported, but its precise functional contributions and mechanisms remain incompletely defined. In this study, we aimed to investigate the oncogenic role of GDF15 in CRC. We found that GDF15 is significantly upregulated in CRC tissues and promotes tumor cell proliferation, migration, invasion, and epithelial‐mesenchymal transition (EMT). Moreover, we provide novel evidence that GDF15 enhances cancer stemness properties. To evaluate its clinical relevance, we further analyzed the correlation between serum GDF15 levels and key clinicopathological parameters in CRC patients. These results may provide a basis for future research on (CRC). These findings elucidate a multifaceted pro‐tumorigenic role for GDF15 in CRC and suggest its potential utility as a biomarker and therapeutic target.

## 2. Materials and Methods

### 2.1. Cell Culture

All cell lines were cultured in Dulbecco’s Modified Eagle’s Medium (DMEM; Catalog No. KGL1206‐500, KeyGEN BioTECH, Nanjing, China) supplemented with 10% certified Fetal Bovine Serum (FBS; Catalog No. FB2500, VICMED, Xuzhou, China). Polyethylenimine (PEI; HY‐K2014, MCE, USA) was used to transfect the cells.

### 2.2. Western Blot, Antibodies and Reagents

The extraction of cellular proteins was carried out using RIPA lysis buffer (supplemented with protease inhibitors) on ice for 30 min, followed by centrifugation at 12,000 × *g* for 15 min at 4°C to collect the supernatant. Protein concentrations were determined using a BCA assay kit. Subsequently, they were separated by sodium dodecyl sulfate‐polyacrylamide gel electrophoresis (SDS‐PAGE), and then transferred onto a polyvinylidene fluoride membrane (Millipore). The blotting was detected using the indicated primary antibody, and then the secondary antibody conjugated with IgG and peroxidase was used for detection. The following antibodies were used in this study: rabbit anti‐Flag (20543‐1), mouse anti GAPDH (60004‐1), goat anti mouse IgG‐HRP, and goat anti‐rabbit IgG‐HRP were from Proteintech.

### 2.3. Colony Formation Assay

The cells were seeded in 3.5 cm culture dishes, with 500–1000 cells per well. The cultures were incubated for 2 weeks until colonies became visible. After gentle washing with PBS, the cells were stained with crystal violet for half an hour. A 0.1% crystal violet solution (25% methanol) was prepared. The formula for calculating the colony formation efficiency was colony formation number/number of seeded cells × 100%.

### 2.4. Cell Proliferation Assay (CCK‐8)

Cells were seeded in 96‐well plates. At indicated time points, 10 μL of Cell Counting Kit‐8 reagent (CCK‐8; Catalog No. VC5001L, VICMED, Xuzhou, China) was added to each well, followed by incubation for 2 h. Absorbance was measured at 450 nm using a microplate reader.

### 2.5. Tissue Specimens

Paired CRC and adjacent normal tissues, located away from the tumor border, were collected from 12 patients at the Xuzhou Central Hospital. The resected fresh tissue samples were immediately frozen in liquid nitrogen, and histological and pathological examinations and grading of each specimen were conducted by two experienced pathologists. Informed consent was obtained from all patients before treatment, and the study received approval from the Ethics Committee of the Xuzhou Central Hospital.

### 2.6. Transwell and Wound‐Healing Assay

For the migration assay, CRC cells were seeded in Transwell plates (3422, Corning, Corning, NY, USA) at a density of 1 × 10^4^ cells per well. After a certain period of cultivation, the cells were fixed with 4% paraformaldehyde and stained with 0.1% crystal violet. For the invasion assay, the cells were seeded in the upper chamber coated with Matrigel, following the same procedure as the migration experiment. The transduced CRC cells were cultured in 6‐well plates and fused. A straight scratch was made using a 10 μL pipette tip. Images of the same area were collected at different time points. The gap was measured at two time points to calculate the migration ability.

### 2.7. Tumorsphere Formation Assay

Tumor cells in the logarithmic growth phase were trypsinized to generate a single‐cell suspension. Cells were resuspended in serum‐free sphere‐forming medium (DMEM/F12 containing 2% B‐27 supplement, 20 ng/mL EGF, 20 ng/mL bFGF, and 1% penicillin‐streptomycin). After counting, cells were seeded at a density of 5000 cells/well into ultra‐low attachment 6‐well plates (Corning, USA) and cultured for 10 days under standard conditions (37°C, 5% CO_2_). To maintain sphere growth, half of the medium was carefully replaced with fresh sphere‐forming medium every other day. After the incubation period, spheres with a diameter >50 μm were imaged and counted using an inverted microscope.

### 2.8. Clinical Data and Serum GDF15 Analysis

A total of 88 CRC patients diagnosed at Xuzhou Central Hospital were included in the clinical correlation analysis. Peripheral blood samples were collected prior to any treatment. Serum levels of GDF15 were quantified using a commercially available enzyme‐linked immunosorbent assay (ELISA) kit (Jianglai Biotechnology, Shanghai, China, JL19786‐96T) according to the manufacturer‘s instructions. Patients were dichotomized into high and low GDF15 groups based on the median serum concentration. Clinical and pathological parameters, including age, gender, TNM stage (AJCC 8th edition), differentiation, lymph node status, tumor size, and levels of carcinoembryonic antigen (CEA) and CA72‐4, were retrieved from medical records. The correlations between serum GDF15 levels (high vs. low) and clinicopathological variables were analyzed using the chi‐square test or Fisher’s exact test, as appropriate. A two‐tailed *p*‐value < 0.05 was considered statistically significant.

### 2.9. Statistical Analysis

Quantitative data are presented as the mean ± standard deviation (SD). Statistical analyses and graph generation were performed using GraphPad Prism software (version 8.0). For comparisons between two groups, an unpaired two‐tailed Student’s *t*‐test was employed. A *p*‐value of less than 0.05 was considered statistically significant, and significance levels are denoted as follows:  ^∗^ *p* < 0.05,  ^∗∗^ *p* < 0.01,  ^∗∗∗^ *p* < 0.001,  ^∗∗∗∗^ *p* < 0.0001; ns, not significant.

## 3. Results

### 3.1. GDF15 is Overexpressed in CRC and Correlates With Aggressive Clinicopathological Features

To evaluate GDF15 expression in CRC, we first analyzed public transcriptomic and proteomic databases. Both mRNA and protein levels of GDF15 were significantly elevated in CRC tissues compared to normal controls (Figure 1A–M). Consistent with bioinformatics analysis, Western blot validation using paired clinical samples from 10 CRC patients confirmed a marked increase in GDF15 protein expression in tumor tissues versus adjacent non‐tumor tissues (Figure 1L). These results indicate that the expression of GDF15 is elevated in CRC. To further investigate the clinical relevance of GDF15 overexpression, we analyzed the correlation between serum GDF15 levels and clinicopathological parameters in 88 CRC patients (Table 1). Patients were stratified into high and low GDF15 groups based on the median serum level. Notably, high serum GDF15 levels were significantly associated with advanced age (>65 years, *p* = 0.001), advanced TNM stage (Stage III/IV, *p* = 0.002), and elevated carcinoembryonic antigen (CEA ≥5 ng/mL, *p* = 0.006). No significant correlations were found with gender, tumor differentiation, lymph node infiltration, tumor diameter, or CA72‐4 levels. These clinical correlations underscore the potential of GDF15 as a biomarker associated with more aggressive disease features in CRC.

Figure 1The expression of GDF15 in CRC patients. (A–K) The mRNA expression of GDF15 in the database. (L) The protein expression of GDF15 in the database. (M) The protein expression of GDF15 in CRC patients.

**Figure:**
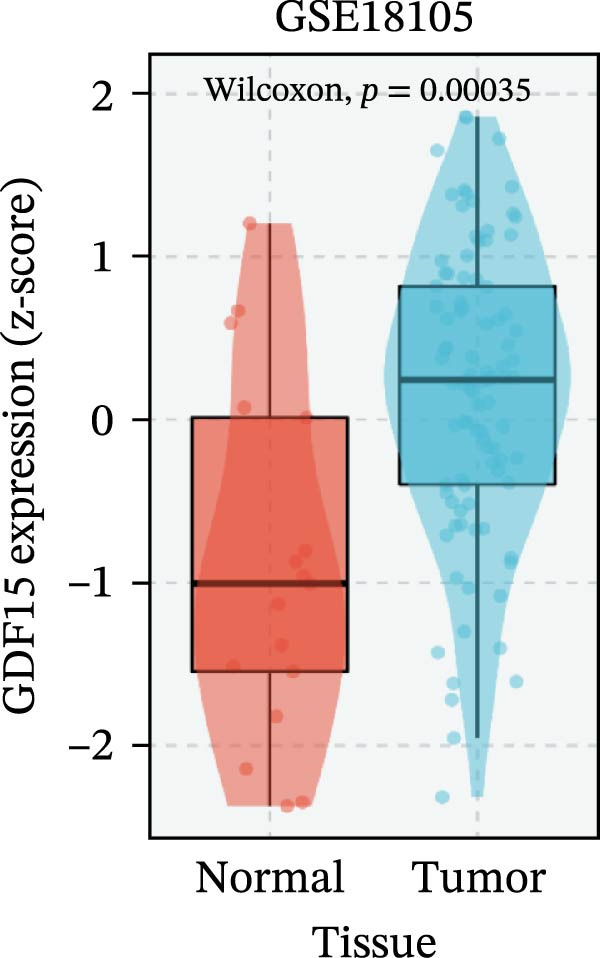
(A)

**Figure:**
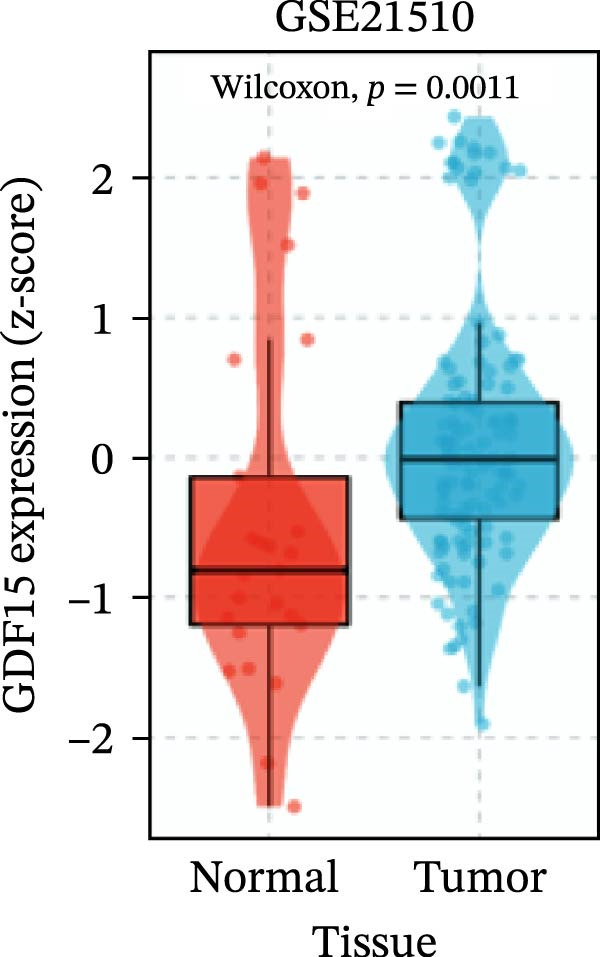
(B)

**Figure:**
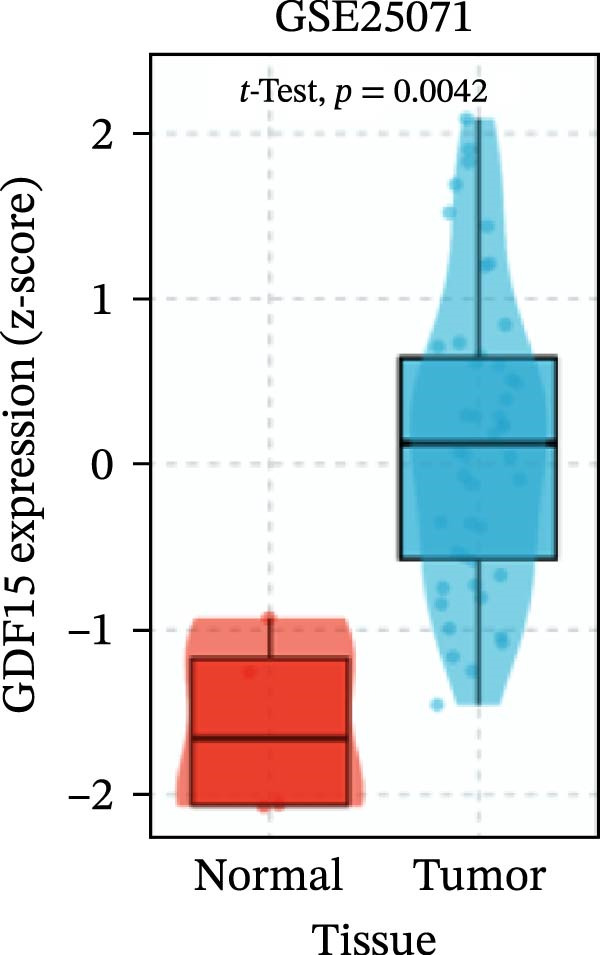
(C)

**Figure:**
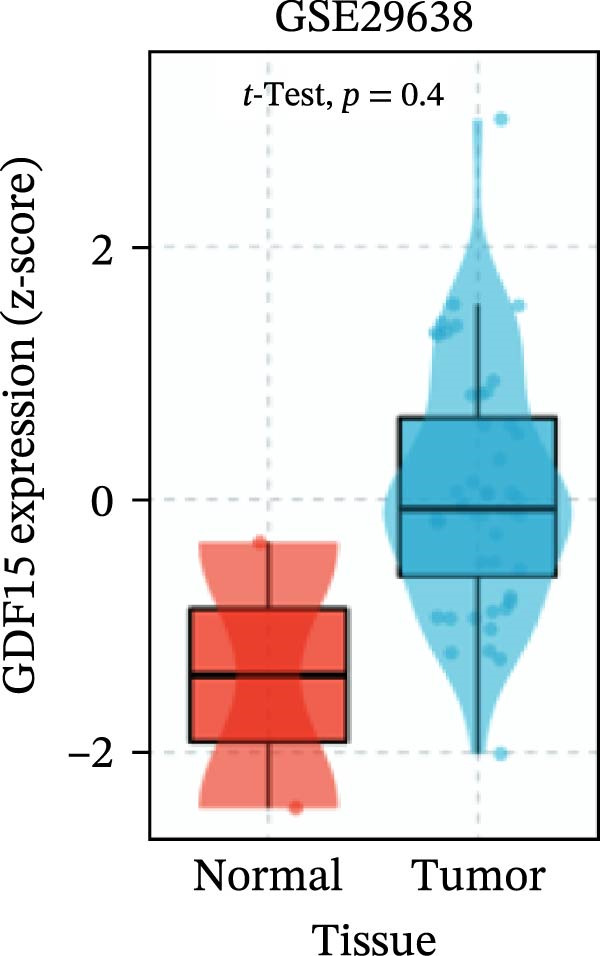
(D)

**Figure:**
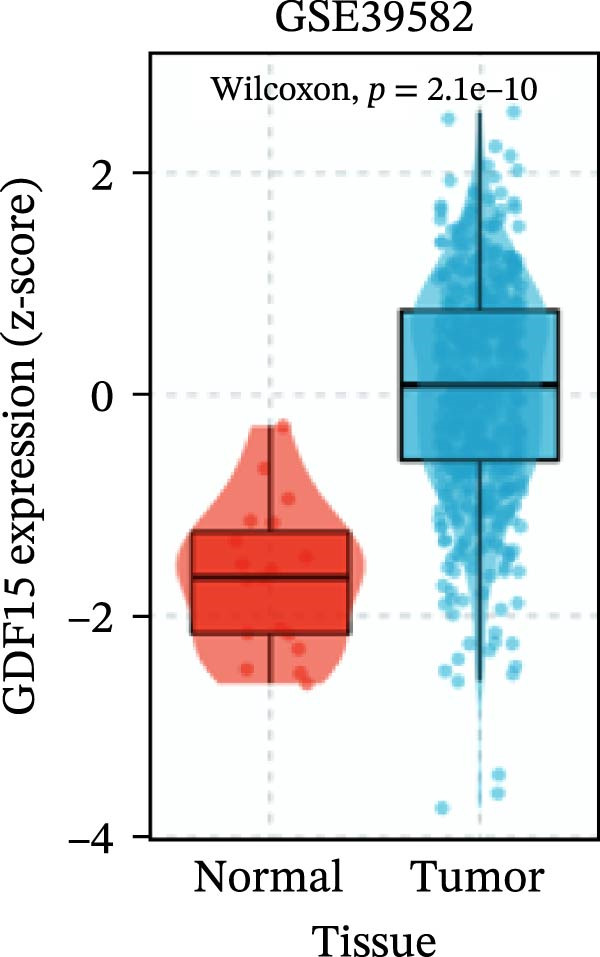
(E)

**Figure:**
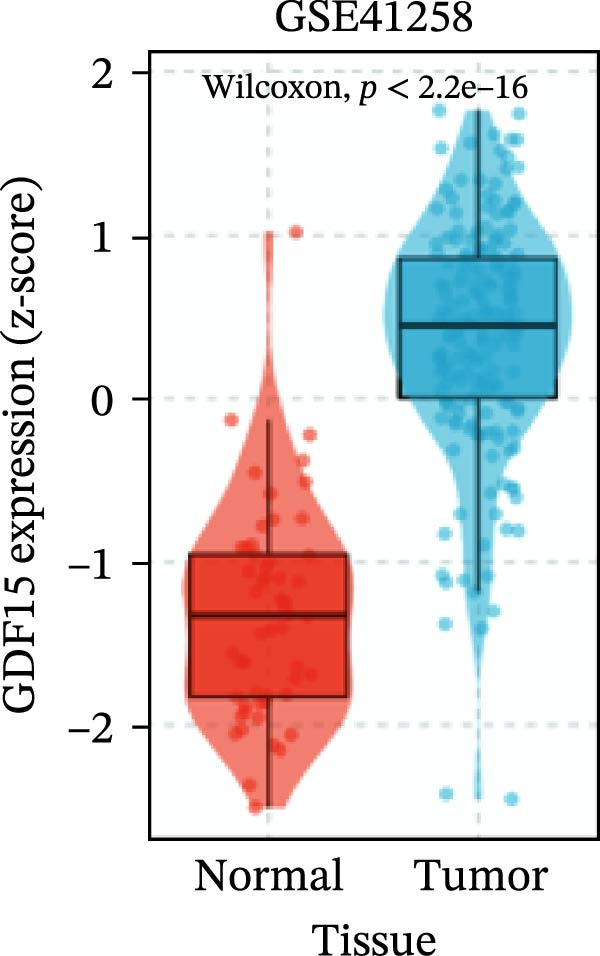
(F)

**Figure:**
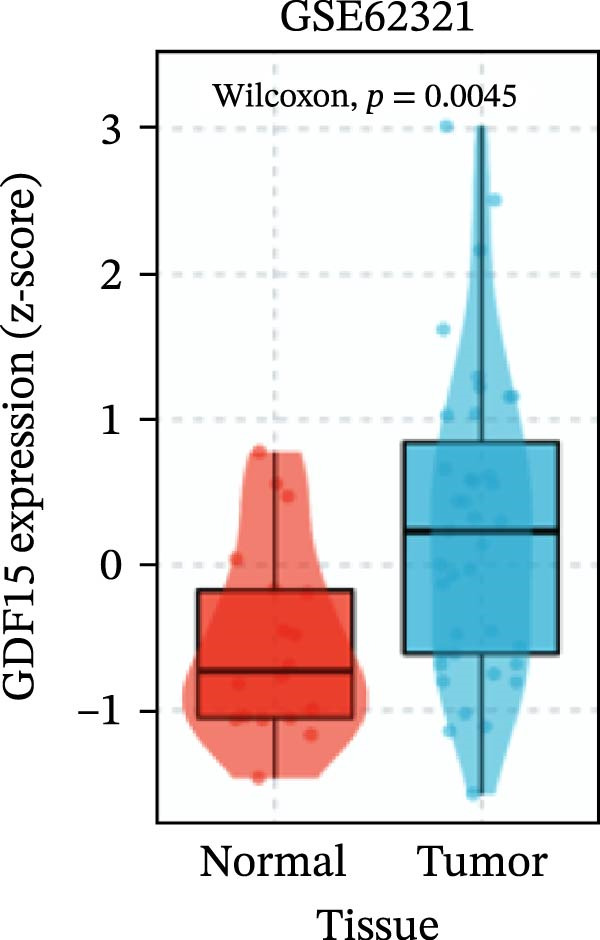
(G)

**Figure:**
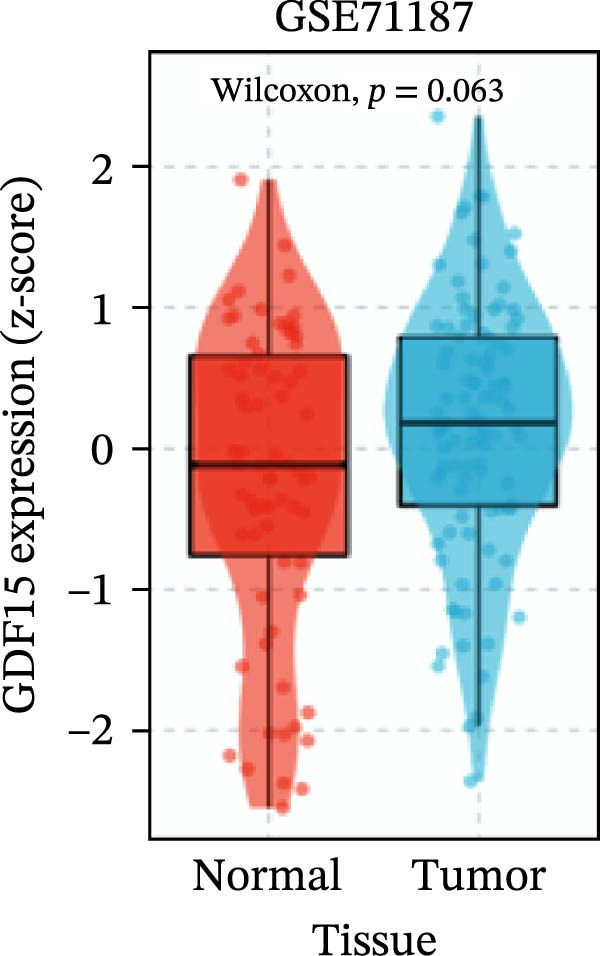
(H)

**Figure:**
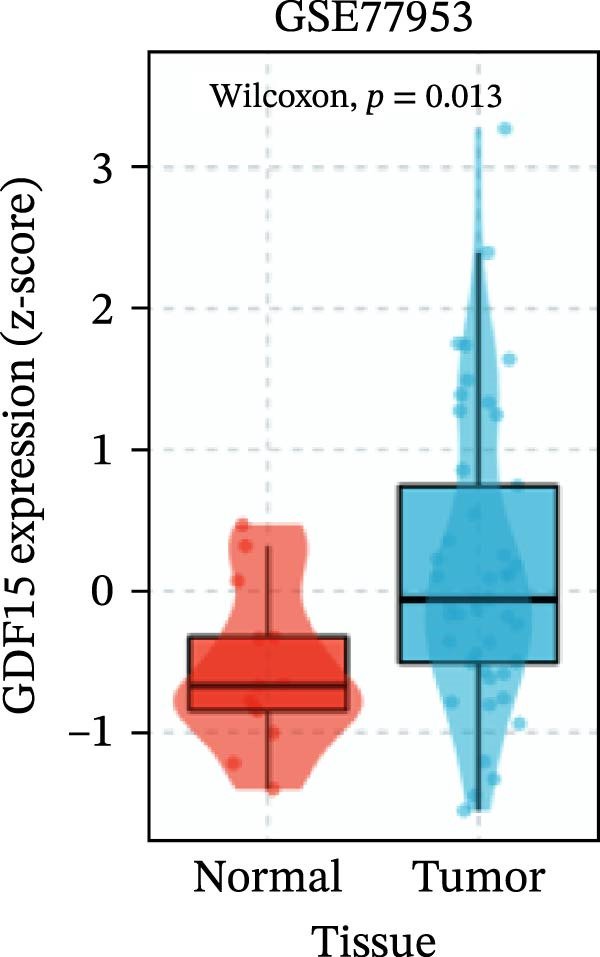
(I)

**Figure:**
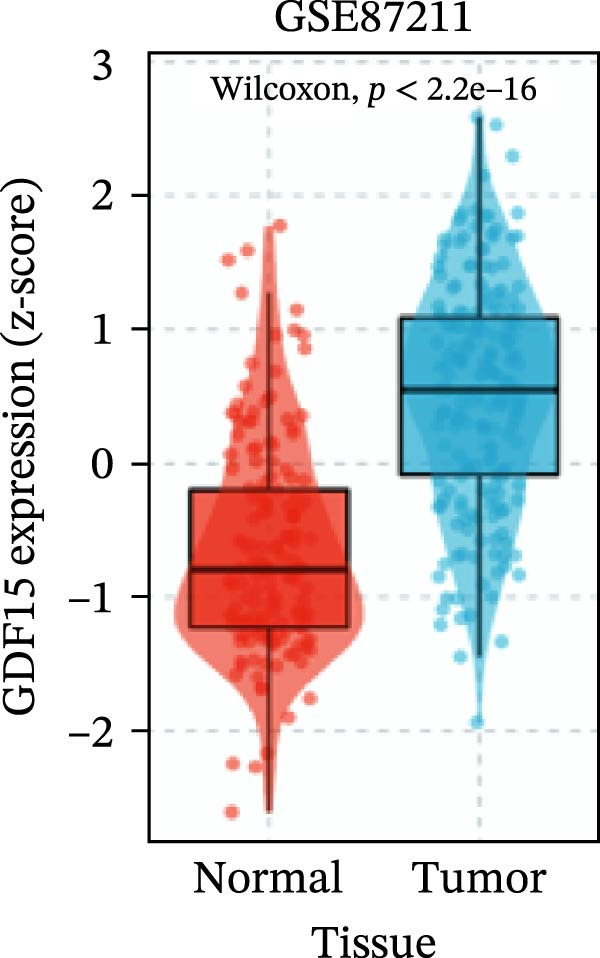
(J)

**Figure:**
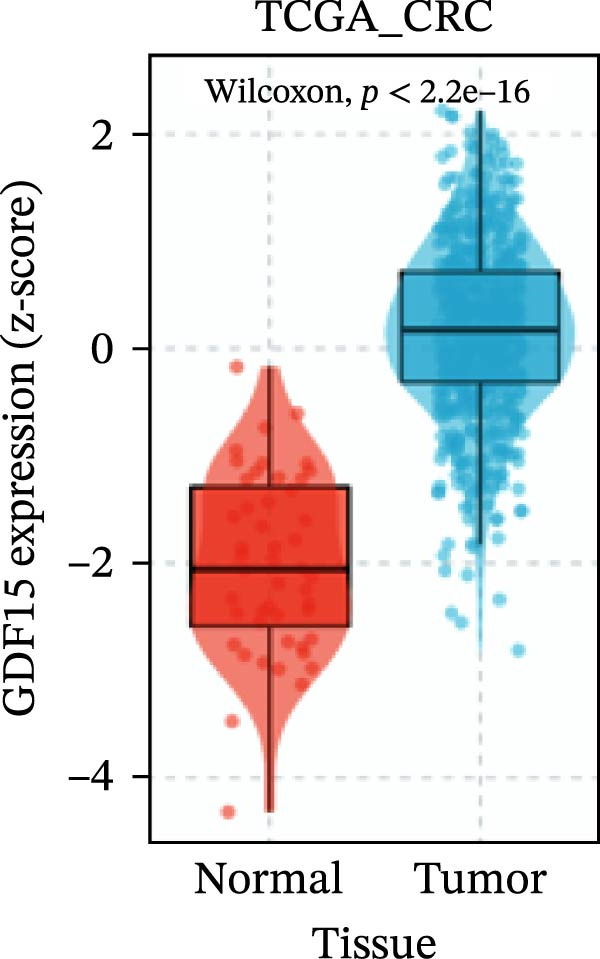
(K)

**Figure:**
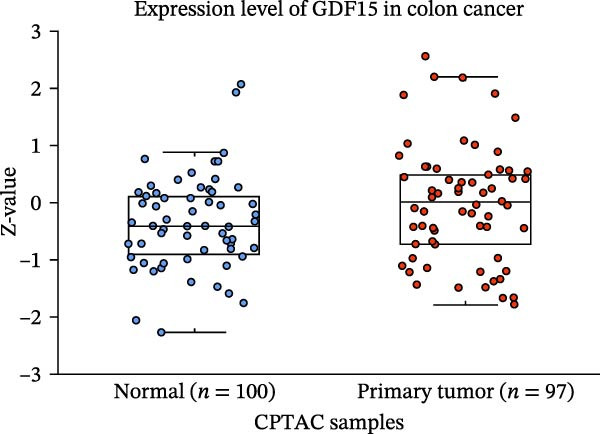
(L)

**Figure:**
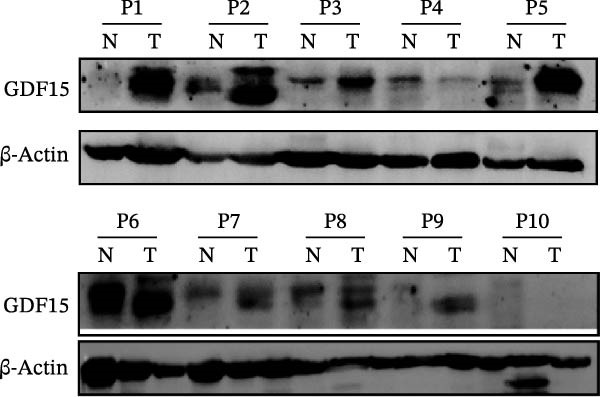
(M)

**Table 1:** The correlation between serum GDF15 levels and clinical pathological parameters.

Characteristics	*n*	High GDF15		Low GDF15	*p*‐Value
		(*n* = 44)		(*n* = 44)	
Age (years)	—	—	—	—	—
＞65	44	30	—	14	**0.001**
≤65	44	14	—	30	—
Gender	—	—	—	—	—
Male	55	32	—	23	**0.048**
Female	33	12	—	21	—
Differentiation	—	—	—	—	—
Well and moderate	57	30	—	27	0.503
Poor	31	14	—	17	—
TNM stage	—	—	—	—	—
I/II	32	9	—	23	**0.002**
III/IV	56	35	—	21	—
Lymph nodeinfiltration	—	—	—	—	—
Yes	31	17	—	15	0.202
No	52	25	—	27	—
Tumor diameter (cm)	—	—	—	—	—
≥5	28	16	—	12	0.361
＜5	60	28	—	32	—
CEA(ng/mL)	—	—	—	—	—
≥5	43	28	—	15	**0.006**
＜5	45	16	—	29	—
CA72‐4 (U/mL)	—	—	—	—	—
≥7	37	21	—	16	0.281
＜7	51	23	—	28	—

*Note:* Values in bold indicate statistically significant differences (*p* < 0.05).

### 3.2. GDF15 Promotes EMT in CRC Cells

Given the association between GDF15 and cancer progression, we investigated its role in EMT, a key process driving metastasis. Overexpression of GDF15 in LOVO cells led to a mesenchymal morphological shift and significantly downregulated the epithelial marker E‐cadherin while upregulating the mesenchymal markers imentin and N‐cadherin (Figure 2A). Conversely, knockdown of GDF15 in HCT116 cells resulted in an epithelial phenotype with increased E‐cadherin and decreased vimentin/N‐cadherin expression (Figure 2B). These results indicate that GDF15 is a potent inducer of EMT in CRC cells.

Figure 2The expression of GDF15 affects the EMT of CRC cells. (A, B) Expression of EMT markers in LOVO and HCT116 cells.

**Figure:**
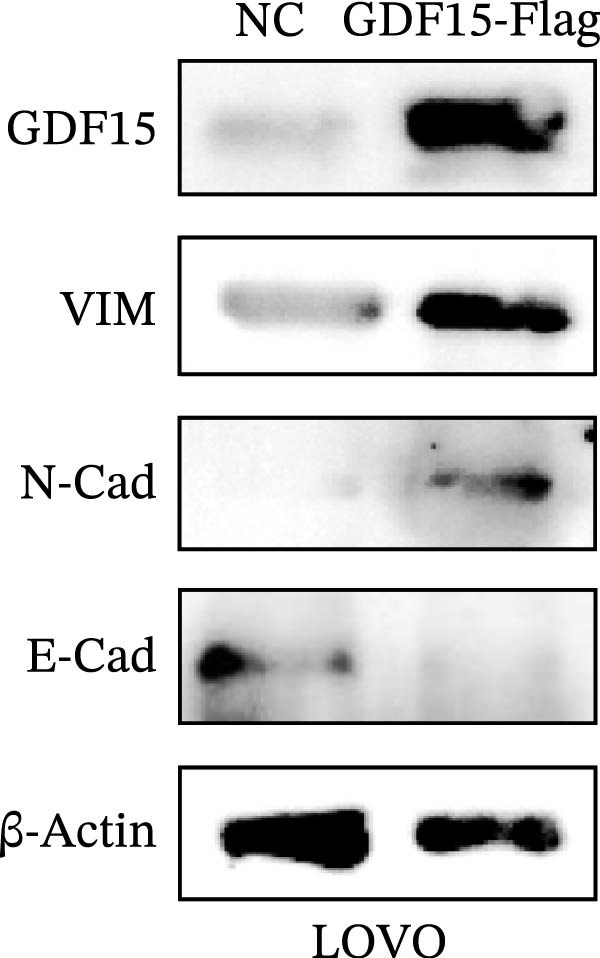
(A)

**Figure:**
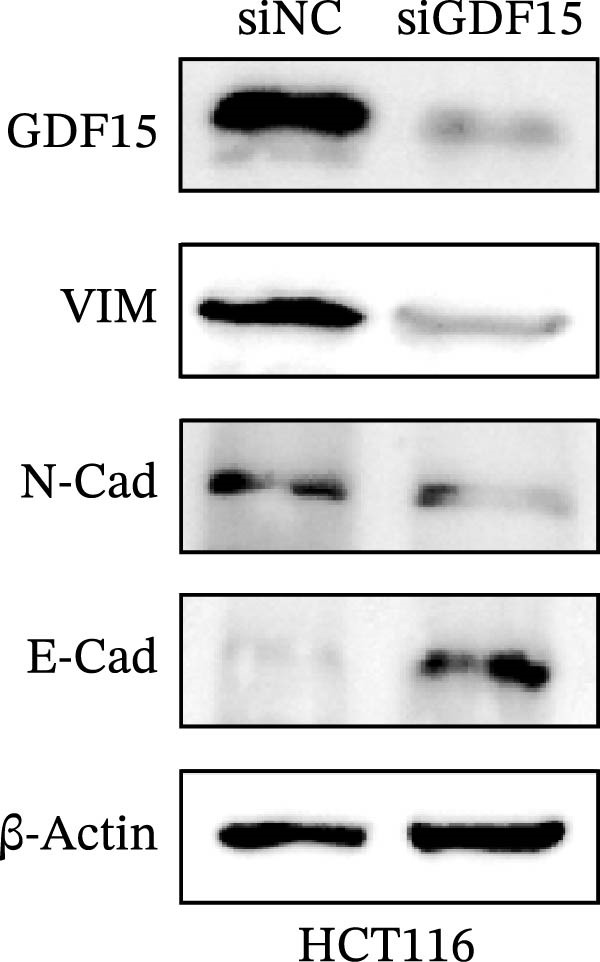
(B)

### 3.3. GDF15 Promotes the Proliferation, and Migration of CRC Cells

We next examined the functional impact of GDF15 on malignant behaviors. Colony formation and CCK‐8 assays demonstrated that GDF15 overexpression significantly enhanced the proliferative capacity of LOVO cells, whereas its knockdown inhibited the growth of HCT116 cells (Figure 3A–D). Furthermore, we examined whether GDF15 affects the metastasis and invasion of CRC cells. We conducted wound‐healing and Transwell assays. The results indicate that overexpression of GDF15 in LOVO cells can enhance their migratory ability. Knockdown of GDF15 in HCT116 cells leads to the opposite result (Figure 3E–H). Western blot analysis further revealed that GDF15 upregulated the proliferation marker PCNA and the metastasis‐associated matrix metalloproteinases MMP2 and MMP9 (Figure 3I, J). Collectively, these findings demonstrate that GDF15 drives both proliferation and metastasis in CRC.

Figure 3GDF15 affects the proliferation and metastasis of CRC cells. (A–D) Clonogenic assay and CCK8‐ assay of CRC cells with GDF15 overexpression and knockdown. (E, F) Alterations in wound‐healing capacity and (G, H) cell migration within tumor cells overexpressing and knockdowning GDF15. (I, J) Western blot analysis of PCNA, MMP9, and MMP2 protein expression in CRC cells with GDF15 overexpression and knockdown.

**Figure:**
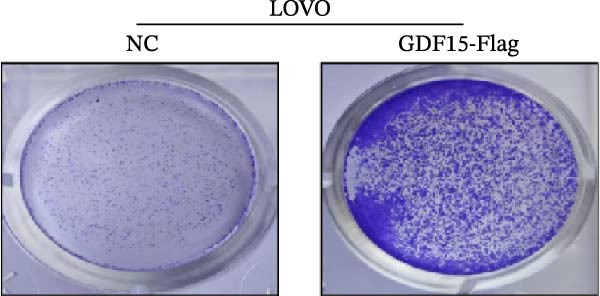
(A)

**Figure:**
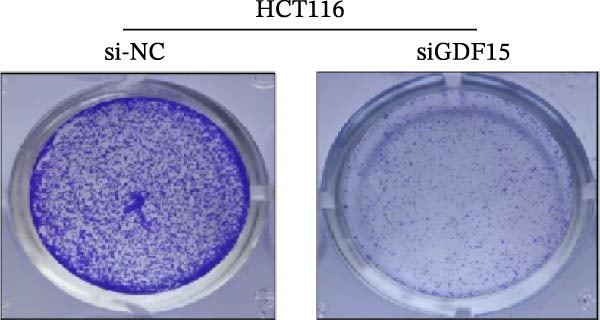
(B)

**Figure:**
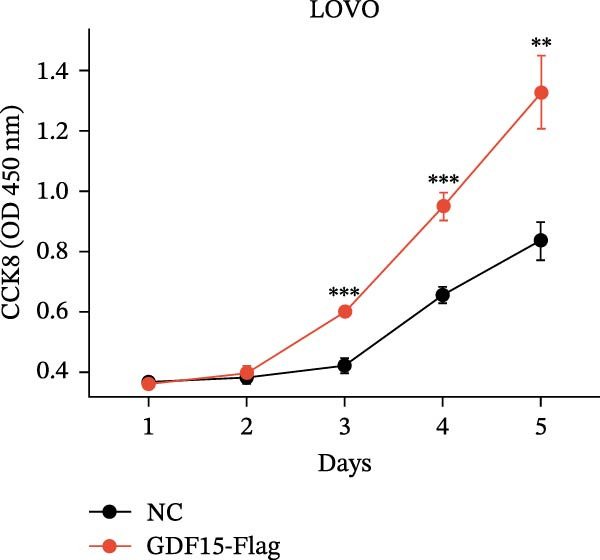
(C)

**Figure:**
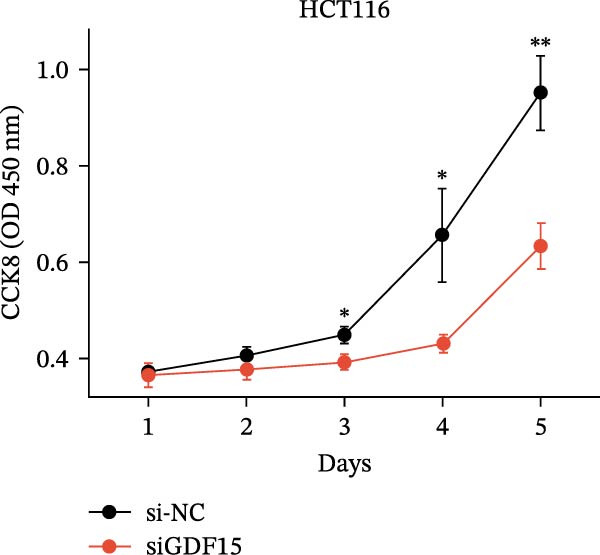
(D)

**Figure:**
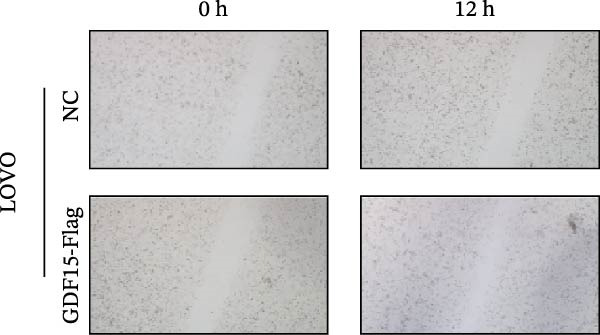
(E)

**Figure:**
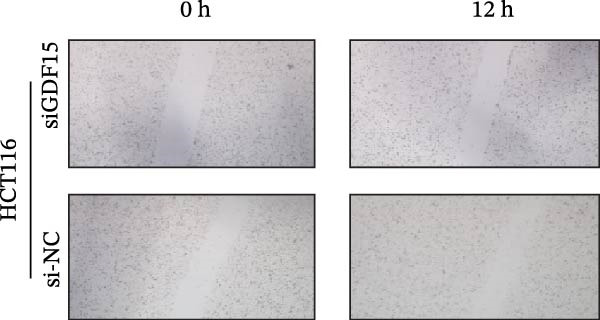
(F)

**Figure:**
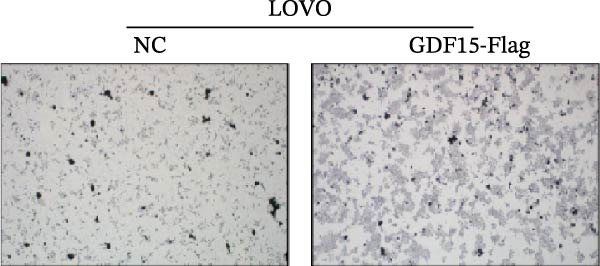
(G)

**Figure:**
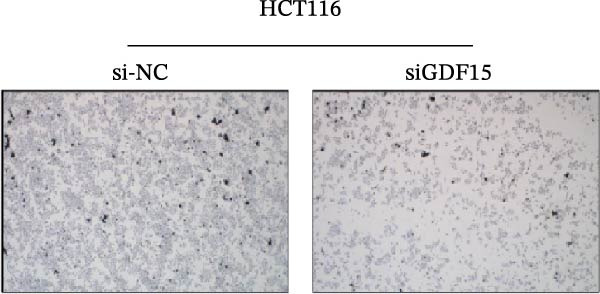
(H)

**Figure:**
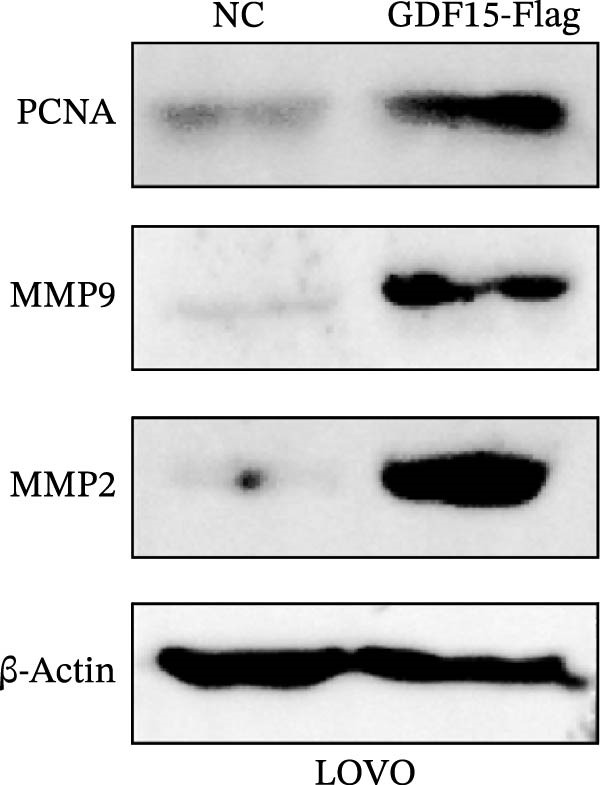
(I)

**Figure:**
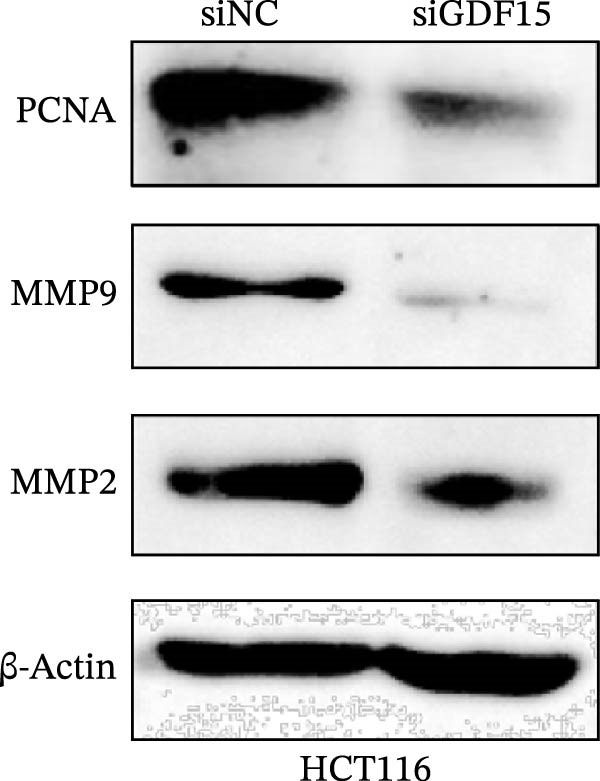
(J)

### 3.4. GDF15 Promotes Cancer Stemness Properties of CRC

Cancer stem cells (CSCs) are critical for tumor initiation, progression, and therapy resistance [19, 20]. To determine whether GDF15 influences CSC traits, we performed tumorsphere formation assays. GDF15‐overexpressing LOVO cells formed more numerous and larger tumorspheres under non‐adherent conditions, while GDF15 knockdown in HCT116 cells suppressed sphere‐forming capacity (Figure 4A, B). To further confirm the role of GDF15 in regulating the stemness process of CRC cancer cells, we examined the protein expressions of tumor stemness‐related markers CD133, SALL4, OCT4, and NANOG. The results showed that the expression of these proteins was elevated upon GDF15 overexpression and reduced upon its knockdown (Figure 4C,D). These data strongly suggest that GDF15 enhances the self‐renewal and stemness properties of CRC cells.

Figure 4GDF15 promotes cancer stemness properties of CRC. (A, B) Tumorsphere formation assay of CRC cells with GDF15 overexpression and knockdown. (C, D) Western blot analysis of the expression of stemness‐related proteins in CRC cells with GDF15 overexpression and knockdown.

**Figure:**
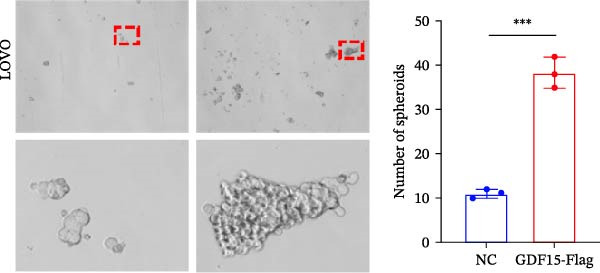
(A)

**Figure:**
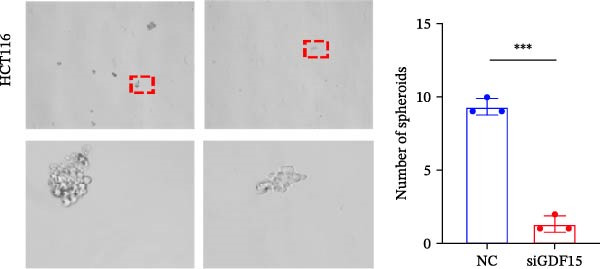
(B)

**Figure:**
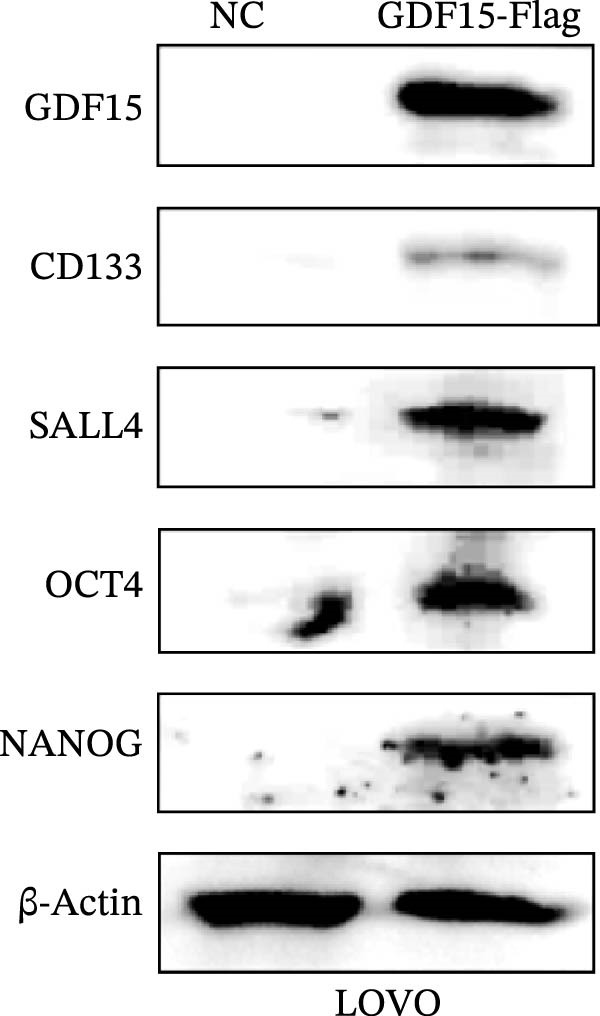
(C)

**Figure:**
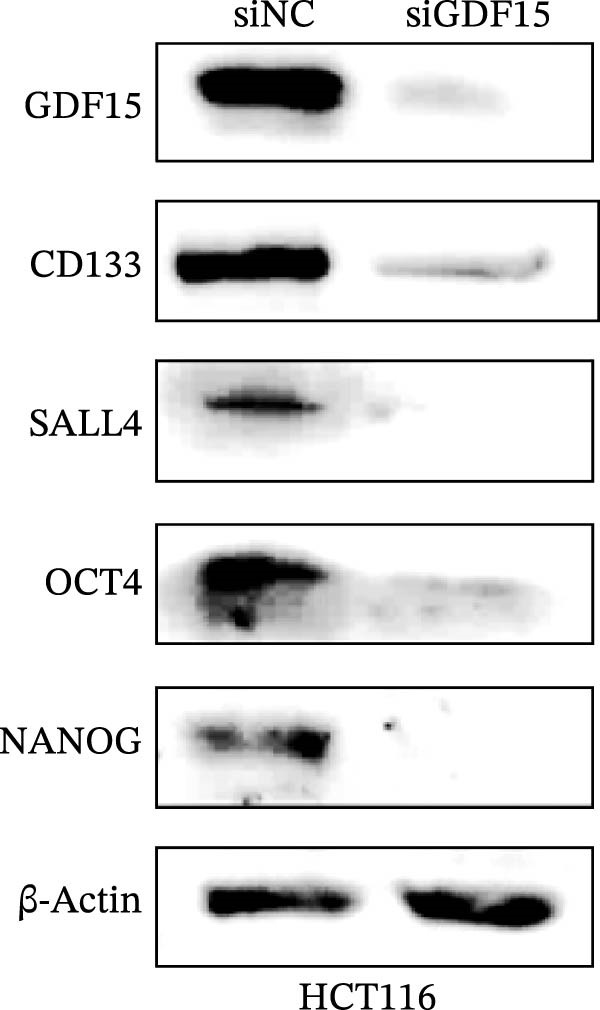
(D)

## 4. Discussion

GDF15 has emerged as a multifaceted cytokine implicated in various diseases, including metabolic disorders [7, 21], liver diseases [22], and pediatric diseases [23]. GDF15 has also been reported to be associated with the progression of various tumors. For instance, studies have shown that GDF15 is upregulated in HCC tissues and inhibits apoptosis of HCC cells. Moreover, its role in promoting the development of HCC and immune evasion is achieved by suppressing the immune response in the tumor microenvironment [24–26]. In recent studies, it has been found that GDF15 promotes the advancement and migration of pancreatic cancer cells. Moreover, in the pancreatic adenocarcinoma microenvironment, solid stress activates the Akt/CREB1 pathway, which upregulates the expression of GDF15 and promotes the metastasis of pancreatic cancer cells. Further research has shown that in advanced pancreatic cancer patients in the high GDF15 group, the levels of phosphorylated‐Jun N‐terminal kinase and Akt increase, highlighting the involvement and regulatory role of GDF15 [27–29]. In gastric cancer, the expression of GDF15 increases and activates the STAT3 signaling pathway, thereby triggering its proliferation and migration. Additionally, GDF15 induces cisplatin resistance in gastric cancer by activating the GFRAL‐GCN2 signaling pathway. Therefore, GDF15 is considered a potential biomarker for the diagnosis and treatment of gastric cancer [30–32]. Our findings align with and extend previous reports. Consistent with studies in pancreatic and gastric cancers. Notably, we identified a novel function of GDF15 in enhancing cancer stemness. GDF15 increased tumorsphere formation and upregulated a panel of core stemness transcription factors (CD133, SALL4, OCT4, NANOG). This suggests that GDF15 not only fuels tumor growth and dissemination but may also contribute to therapy resistance and recurrence by enriching the CSC pool, a hypothesis warranting future investigation.

The precise signaling mechanisms downstream of GDF15 in CRC require further elucidation. While GFRAL is its canonical receptor in the brain, its expression and role in peripheral tumors are less clear. GDF15 may activate alternative receptors or signaling pathways in cancer cells, such as TGF‐β receptors or integrin‐mediated pathways, leading to the activation of SMAD, PI3K/Akt, or MAPK cascades. Future studies should delineate the receptor usage and key downstream effectors mediating GDF15’s effects on EMT and stemness in CRC.

Our study has limitations. The conclusions are primarily based on in vitro experiments using two cell lines. Future work should include in vivo xenograft or genetically engineered mouse models to validate the tumor‐promoting role of GDF15. Additionally, exploring the correlation between serum GDF15 levels, tumor GDF15 expression, and patient clinicopathological features (e.g., stage, metastasis, survival) in larger cohorts would strengthen its potential as a clinical biomarker.

In conclusion, we demonstrate that GDF15 functions as a key oncogenic driver in CRC by promoting EMT, proliferation, metastasis, and stemness. This is partly consistent with the previous research results [33–35]. These findings position GDF15 as a promising multifaceted target for CRC therapy. Strategies to neutralize GDF15 activity, such as monoclonal antibodies or receptor antagonists, which are under development for metabolic diseases and cachexia, may hold therapeutic potential for a subset of CRC patients with high GDF15 expression.

## Author Contributions

Hui Xu designed the research. Quancheng Zhang and Qing Li were responsible for data collection under the supervision of Hui Xu. Hui Xu, Quancheng Zhang, and Qing Li conceived and planned the experiments and analyzed the data. Hui Xu, Quancheng Zhang, and Qing Li wrote the first draft of the manuscript, and Feng Gu, Duping Wang, and Yiqing Tian critically reviewed and revised the manuscript for important intellectual content, and it was also improved by all authors. Hui Xu, Quancheng Zhang, and Qing Li were responsible for the final content.

## Funding

This project is supported by Jiangsu Key Laboratory of Immunity and Metabolism (Grant XZSYSKF2022040).

## Disclosure

The authors have read and approved and take full responsibility for the final content of the publication of the manuscript.

## Conflicts of Interest

The authors declare no conflicts of interest

## Data Availability

The data that support the findings of this study are available from the corresponding author upon reasonable request.
